# ANGPTL8 deletion attenuates abdominal aortic aneurysm formation in *ApoE*^−/−^ mice

**DOI:** 10.1042/CS20230031

**Published:** 2023-06-28

**Authors:** Huahui Yu, Xiaolu Jiao, Yunyun Yang, Qianwen Lv, Zhiyong Du, Linyi Li, Chaowei Hu, Yunhui Du, Jing Zhang, Fan Li, Qiuju Sun, Yu Wang, Dong Chen, Xiaoping Zhang, Yanwen Qin

**Affiliations:** 1 The Key Laboratory of Remodeling-Related Cardiovascular Diseases, Ministry of Education, National Clinical Research Center for Cardiovascular Diseases, Beijing Institute of Heart Lung and Blood Vessel Disease, Beijing Anzhen Hospital, Capital Medical University, Beijing 100029, China; 2 Department of Pathology, Beijing AnZhen Hospital, Capital Medical University, Beijing 100029, China

**Keywords:** Abdominal aortic aneurysm, Angiopoietin-Like Protein 8, inflammation

## Abstract

Angiopoietin-like protein 8 (ANGPTL8) plays important roles in lipid metabolism, glucose metabolism, inflammation, and cell proliferation and migration. Clinical studies have indicated that circulating ANGPTL8 levels are increased in patients with thoracic aortic dissection (TAD). TAD shares several risk factors with abdominal aortic aneurysm (AAA). However, the role of ANGPTL8 in AAA pathogenesis has never been investigated. Here, we investigated the effect of ANGPTL8 knockout on AAA in *ApoE^−/−^* mice. *ApoE^−/−^ANGPTL8^−/−^* mice were generated by crossing *ANGPTL8^−/−^* and *ApoE^−/−^* mice. AAA was induced in *ApoE^−/−^* using perfusion of angiotensin II (AngII). ANGPTL8 was significantly up-regulated in AAA tissues of human and experimental mice. Knockout of ANGPTL8 significantly reduced AngII-induced AAA formation, elastin breaks, aortic inflammatory cytokines, matrix metalloproteinase expression, and smooth muscle cell apoptosis in *ApoE^−/−^* mice. Similarly, ANGPTL8 sh-RNA significantly reduced AngII-induced AAA formation in *ApoE^−/−^* mice. ANGPTL8 deficiency inhibited AAA formation, and ANGPTL8 may therefore be a potential therapeutic target for AAA.

## Introduction

Abdominal aortic aneurysm (AAA) is a life-threatening cardiovascular disease (CVD) caused in part by atherosclerosis and characterized by endothelial dysfunction, lipid accumulation, and inflammatory cell infiltration of the arterial wall [1]. AAA is defined as a focal dilation of the aortic diameter ≥3 cm or exceeding the normal vascular diameter by more than 50% [2]. Effective pharmacological therapies to inhibit AAA expansion or prevent aneurysm rupture have not been established clinically [3], and the molecular pathogenesis of AAA remains unclear [4]. There is an urgent clinical need to find targets to inhibit the development of AAA.

Angiopoietin-like protein 8 (ANGPTL8) is a protein that belongs to the family of angiopoietin-like proteins [5]. Under normal physiological conditions, ANGPTL8 is secreted by the liver and adipose tissue [6,7]. Resent reports have shown that ANGPTL8 is highly expressed in cell of arteries in response to the stimulation by a number of pathological factors, including AngII [8]. In addition to being involved in glucose and lipid metabolism, ANGPTL8 also regulates many physiological processes, including inflammation [9], oxidative stress [10], cell proliferation and apoptosis [11,12], which are risk factors for the development of CVD. ANGPTL8 has been reported to be associated with CVD [13]. But the role of ANGPTL8 in the AAA has not been demonstrated.

Therefore, in the present study, we investigated the role of ANGPTL8 in AAA and atherosclerosis using *ApoE*^−/−^*ANGPTL8*^−/−^ double-knockout mice.

## Methods

### Human tissues

Surgical aortic aneurysm specimens were obtained from patients with AAA underwent open surgical repair at Beijing An Zhen Hospital affiliated with Capital Medical University (Beijing, China). Diagnostic criteria for AAA were based on the latest diagnostic standard [14]. Human atherosclerotic lesions were collected from patients underwent carotid endarterectomy at Beijing An Zhen Hospital. Control aortic specimens were obtained from healthy heart transplant donors in Beijing An Zhen Hospital, those with collagen disease or atherosclerotic disease were excluded as previously described [15]. Informed consent was obtained from all patients according to the protocol approved by the Medical Ethical Committee of Beijing An Zhen Hospital in compliance with the principles of the Declaration of Helsinki. Tissue specimens were fixed in 10% formalin, embedded in paraffin, and sectioned at 5-μm thick (*n*=6).

### Human plasma

The study included a total of 80 subjects, including 40 patients with AAA and 40 controls. Subjects with AAA were defined as computed tomographic angiography (CTA) to assess AAA morphometry with an AAA diameter of >30 mm. A total of 40 volunteers from the Health Examination Center at Beijing An Zhen Hospital were enrolled as controls. Control patients were enrolled only if the CT revealed no evidence of aortic diseases. Maximum axial aortic diameters in the abdominal aorta were assessed by CT. We excluded patients with secondary forms of hypertension, liver disease, hepatitis, liver enzyme abnormalities, renal inadequacy, cancer, acute infectious diseases, low life expectancy, and those who had experienced a stroke, acute coronary syndrome, cardiac failure, or peripheral arterial disease. Blood samples were collected from patients during routine preoperative testing for the measurement of plasma ANGPTL8 levels. The study was approved by the Medicine Ethics Committee of Beijing An Zhen Hospital and adhered to the Declaration of Helsinki. All participants gave written informed consent before enrollment.

### ELISA

The levels of circulating ANGPTL8 in patients with and without AAA included in the present study were measured using an ELISA kit (Wuhan ELAAB Science, Wuhan, China; CataloguNo. 11644h). The levels of circulating ANGPTL8 in mice were measured using an ELISA kit (Ruixin Biotech). ELISA was performed using ELISA kits according to the manufacturer’ instructions.

### Animals

Animal experiments were approved by the Ethics Committee of Capital Medical University and performed in accordance with the National Institutes of Health Guide for the Care and Use of Laboratory Animals. All animal experiments took place in SPF Animal Laboratory at Beijing Anzhen Hospital.

Male *ApoE*^−/−^ mice on a C57BL/6 background were purchased from Beijing Huafukang Biotechnology (Beijing, China). *ANGPTL8*^−/−^ mice generated by our laboratory on a C57BL/6J background, as previously described [11]. *ApoE*^−/−^*ANGPTL8*^−/−^ mice were generated by crossing the offspring of *ANGPTL8*^+/−^*ApoE*^+/−^ mice, which were mated to their respective homozygous knockouts and genotyped by polymerase chain reaction. All mice in the described experiments were bred at specific pathogen-free conditions at the animal facilities of Beijing Anzhen Hospital affiliated to the Capital Medical University and were housed under standard 12/12 h light/dark conditions.

For the first part of our study, *ApoE*^−/−^ mice (male, aged 8–10 weeks) were randomly divided into the Saline (*ApoE*^−/−^ + Saline, *n*=20) and AngII (*ApoE*^−/−^ + AngII, *n*=20) groups, whereas *ApoE*^−/−^*ANGPTL8*^−/−^ mice (male, aged 8–10 weeks) were randomly divided into Saline (*ApoE*^−/−^*ANGPTL8*^−/−^ + Saline, *n*=20) and AngII (*ApoE*^−/−^*ANGPTL8*^−/−^ + AngII, *n*=20) groups. Saline groups received continuous subcutaneous infusion of saline, while AngII groups received continuous subcutaneous infusion of AngII (1000 ng/kg per min) via an osmotic pump (Alzet MODEL 1007D; DURECT, Cupertino, CA) for 28 days. Mice were anesthetized with 1% sodium pentobarbital (i.p. injection), followed by mini pump implantation. During 28 days AngII infusion, the deceased mice were subjected to an autopsy to determine the cause of death was aneurysm-related.

For the second part of the study, *ApoE*^−/−^ (*n*=10) and *ANGPTL8*^−/−^*ApoE*^−/−^ (*n*=10) mice aged 8–10 weeks were fed a western diet containing 21% fat by weight (0.15% cholesterol and 19.5% casein without sodium cholate) for 12 weeks.

For the third part of the study, *ApoE*^−/−^ mice were randomly divided into Control and sh-ANGPTL8 groups (male, aged 8–10 weeks; *n*=10 per group). Knockdown of ANGPTL8 was achieved by injection with an AAV-9 encoding a short hairpin RNA (shRNA) specifically targeting mouse ANGPTL8. The shRNA sequence was cloned into AAV-9 by Shanghai Genechem (Shanghai, China) using the target sequence TAAGCAGAGCCACCTCTTATG. The sh-ANGPTL8 group mice were transfected with sh‐ANGPTL8 from tail vein injection. After 2 weeks, all mice were operated with osmotic pumps and received a continuous subcutaneous infusion of AngII. During 28 days AngII infusion, the deceased mice were subjected to an autopsy to determine the cause of death was aneurysm-related.

After 12 weeks of feeding with a western diet or 28 days of AngII infusion, all mice were sacrificed with intraperitoneal injection of 1% sodium pentobarbital, and their aortic tissues were removed for further analysis.

### Mouse genotyping

Tail-tip DNA extracted from mice and primers specific for *ANGPTL8* (5′-CCTGAGTTCTAGCAGCGTGAT-3′ and 5′-ACTTGCAAAGGTCACTTCCAG-3′), *ApoE* (5′-GCCTAGCCGAGGGAGAGCCG-3′, 5′-TGTGACTTGGGAGCTCTGCAGC-3′, and 5′-GCCGCCCCGACTGCATCT-3′) were used for PCR screening. PCR was performed under the following conditions. For ANGPTL8: 98°C for 30 s; 35 rounds of 98°C for 10 s, 63°C for 30 s, and 72°C for 90 s; 72°C for 10 min; and hold at 4°C. For ApoE: 94°C for 3 min; 35 rounds of 94°C for 30 s, 68°C for 40 s, and 72°C for 1 min; 72°C for 2 min; and hold at 4°C. PCR products with a predicted size were analyzed by agarose gel (2% wt/vol) electrophoresis and confirmed by Sanger sequencing.

### Measurement of blood pressure in mice

Blood pressure in conscious mice was monitored weekly using a noninvasive tail-cuff system (BP-98A; Softron, Tokyo, Japan), initiated 1 week before AngII infusion and continuing throughout the study. The measurement was conducted once every week at 9–11 a.m. in a quiet room. Before starting measurements, ten preliminary cycles were performed to allow the mice to adjust to the inflating cuff. The blood pressure of each mice was tested three consecutive times to calculate the mean value.

### Plasma biochemical analysis

Blood samples were collected from sacrificed animals by cardiac puncture. Plasma lipid profiles including total triglycerides (TG), total cholesterol (TC), low-density lipoprotein cholesterol (LDL-C) and blood glucose (GLU) levels were determined using enzymatic colorimetric assays (Zhongsheng Beikong Biotechnology and Science, Beijing, China) according to the manufacturer’s instructions.

### Quantification of AAA

Maximum aortic diameter was measured in each mouse before prior to sacrifice via the high-frequency ultrasound imaging system (Vevo2100; Toronto, Canada). Two-dimensional (B-mode) imaging using a 30 MHz linear-array transducer (MS550D) synchronized to the electrocardiographic signal was done. The animals were placed in a supine position on a heated table under inhalation anesthesia with isoflurane (1%). The abdominal cavity was shaved, and a prewarmed ultrasound gel was applied to the area of interest. Longitudinal images of the suprarenal and infrarenal aorta and transverse images at the level of the abdominal aorta between the diaphragm and the outlet of the left renal artery were acquired in the B-mode and M-mode to assess the maximum cross-sectional diameter (during diastole) in real-time for each mouse at each time point [16]. AAA was defined as a 150% increase in diameter as a criterion. In addition, any dissection leading to intramural hematoma (even if the actual dilatation was only 110%) should be accounted for in the AAA mouse model [17].

### Quantification of atherosclerosis

Mouse aortic trees were prepared and stained, and atherosclerotic lesions were quantified. Briefly, the entire aorta from the root to the iliac bifurcation was collected and fixed in formalin-free fixative. After removal of the adventitial fat, the aorta was opened longitudinally, stained with Oil Red O (Sigma-Aldrich, St. Louis, MO, U.S.A.), and pinned down on black wax to expose the intima. The extent of atherosclerosis was determined as the ratio of the enface lesion area to the entire intimal area.

For analysis of plaque lesions in the aortic sinus, the heart and proximal aorta were removed and embedded in optimal cutting temperature compound. Continuous 7-μm thick frozen sections from the middle portion of the ventricle to the aortic arch were collected and stained with Oil Red O and hematoxylin. The lipid-containing area on each section was determined under light microscopy in a blinded fashion. The average lesion area per aorta was calculated from 5 to 10 sections of each aorta and then determined as described previously [15]. The percentage of the aortic plaque area was calculated as the ratio of the aortic intimal plaque area to the vessel wall area.

### Histopathology and immunohistochemistry analysis

Tissues were cut to 5-μm sections on a microtome. All slides were numbered continuously and the same number of slices from each group was subjected to staining.

For immunohistochemical staining, tissue sections were deparaffinized and rehydrated by successive washes of xylene, 100% ethanol, 95% ethanol, 80% ethanol, and water. Following rehydration, sections were permeabilized with 0.01% Triton X-100 in phosphate-buffered saline (PBS), blocked with 10% goat serum, incubated with primary antibodies at 4°C overnight. The following day, sections were incubated with appropriate horseradish peroxidase-conjugated secondary antibodies and counterstained with hematoxylin. The following primary antibodies were used: ANGPTL8 (1:200 dilution; ab180915; Abcam), matrix metalloproteinase 2 (MMP-2; 1:200 dilution; ab37150, Abcam), MMP-9 (1:200 dilution; ab38898, Abcam), cleaved PARP (1:200 dilution; 9929T; Cell Signaling Technology), and cleaved caspase 3 (1:200 dilution; 9929T, Cell Signaling Technology).

Images were obtained with a Ni-U Nikon Upright Microscope equipped with a DS-Ri2 color charged-coupled device (Nikon, Tokyo, Japan). For statistics, at least six images per section were analyzed using ImageJ software (https://imagej.nih.gov). Relative expression was calculated as a percentage of total pixel intensity in each image.

### Double immunofluorescence staining

Tissue sections were subjected to antigen retrieval and blocking as described above, followed by incubation with a rabbit anti-ANGPTL8 antibody (ab180915, Abcam) together with mouse anti-α-SMA (sc-53142, Santa Cruz), anti-MAC-2 (ab278071, Abcam), and anti-CD3 (sc-20047, Santa Cruz) antibodies in PBS containing 0.2% Triton X-100 at 4°C overnight. After three washes with the same buffer, sections were incubated with Alexa Fluor 555-conjugated and Alexa Fluor 488-conjugated antibodies against rat and mouse or rabbit IgG, respectively, for 2 h at room temperature. After washing section, fluorescent images were acquired with a Nikon TS2-S-SM microscope equipped with a Nikon DS-Qi2 camera.

### *In-situ* zymography

OCT-embedded, fresh abdominal aortic cryosections were analyzed with *in-situ* zymography using the MMP fluorogenic substrate DQ-gelatin-FITC (Invitrogen). Cryosections were incubated with 40 μg/ml (in PBS) of the quenched fluorogenic substrate DQ-gelatin-FITC for 1 h at 37°C. The gelatin with a fluorescent tag remained caged (no fluorescence) until the gelatin was cleaved by a gelatinase. The excess fluorogenic substrate was washed with PBS and photographed with Nikon TS2-S-SM microscope. Quantitative analysis of fluorescence intensity was obtained with ImageJ software.

### Cell culture and conditions

Human aortic smooth muscle cells (HASMCs) and RAW 264.7 cells were obtained from the Shanghai Xinyu Biotech co., Ltd. HASMCs and RAW 264.7 were cultured in the DMEM medium (Science cell, CA, U.S.A.) supplemented with 10% fetal bovine serum (FBS) and 1% penicillin/streptomycin solution in a humidified atmosphere containing 5%CO_2/_95% air at 37°C.

### Transfection of ANGPTL8-overexpression vector

We used a lentiviral ANGPTL8-overexpression vector (Shanghai Genechem) to transfect HASMC and RAW 264.7 cells. A Stealth Negative Control Lentiviral Vector (Shanghai Genechem) was used as a control. Stable transfections of the ANGPTL8-overexpression vector were performed according to the manufacturer’s instructions.

### Western blot

Tissues were harvested at the indicated times and homogenized in ice-cold suspension buffer supplemented with a proteinase inhibitor cocktail (Sigma-Aldrich). A protein extraction kit containing protease inhibitors and a protein phosphatase inhibitor cocktail was used to extract protein. Protein concentrations were determined using a BCA Protein Assay Kit (Thermo Fisher Scientific, Waltham, MA, U.S.A.). Subsequently, equal samples were loaded, separated, and transferred to a membrane (Pierce; Thermo Fisher Scientific). Membranes were blocked, incubated overnight at 4°C with the primary antibodies ANGPTL8 (1:1000 dilution; ab180915; Abcam) and β-actin (1:1000 dilution; ab8227, Abcam), washed with Tris-buffered saline containing Tween 20, and incubated with secondary antibodies (1:10000 dilution; ZSGB-BIO) for 1 h at room temperature. Blots were then washed, incubated with SuperSignal™ WestFemto Maximum Sensitivity Substrate (Thermo Fisher Scientific), and analyzed using a ChemiDoc™ Touch Imaging System (Bio-Rad, Hercules, CA, U.S.A.).

### Statistical analysis

Data were analyzed using Prism 8.0 software (GraphPad Software, San Diego, CA, U.S.A.) and are presented as mean ± standard error of the mean (SEM). Each experiment was repeated at least three times. Statistical comparisons between the two groups were performed using Student’s *t*-test. Two-way analysis of variance with Bonferroni’s correction was employed for multiple-comparison tests. Differences between the four groups were tested using ANOVA. The Kaplan–Meier method was used to construct survival curves and the log-rank test was used to determine differences. *P*-values lower than 0.05 were considered statistically significant (*).

## Result

### ANGPTL8 expression was significantly increased in human and mouse AAA tissues

**Figure 1:**
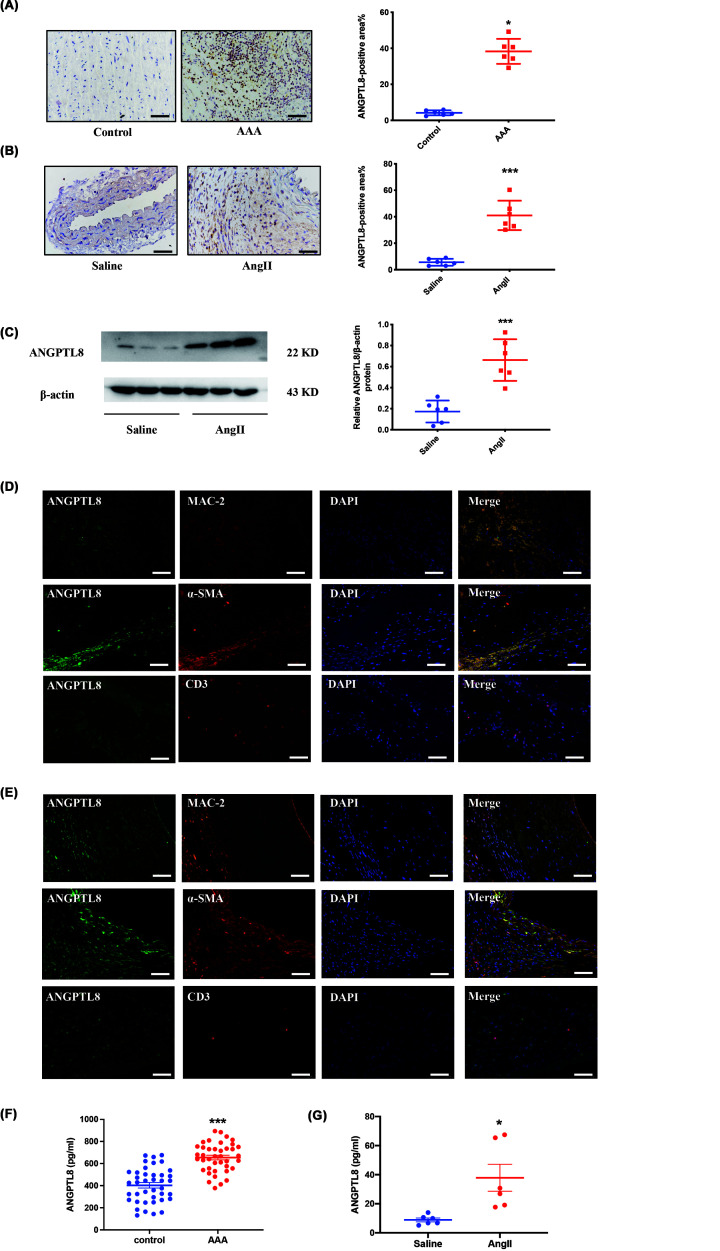
ANGPTL8 expression is significantly up-regulated in human AAA and AngII-induced AAA of *ApoE^−/−^* mice (**A**) Immunohistochemical results of ANGPTL8 in human AAA tissue and control tissues. (**B**) Immunohistochemical results of ANGPTL8 in arterial tissues of saline and AngII-infused AAA in *ApoE^−/−^* mice. (**C**) Western blot results of ANGPTL8 in arterial tissues of saline and AngII-infused *ApoE^−/−^* mice. (**D**) Immunofluorescence analysis of ANGPTL8 (green), MAC-2, α‐SMA, and CD3 (red) and 4′,6‐diamidino‐2‐phenylindole (DAPI; blue for nuclei) in human AAA tissues. (**E**) Immunofluorescence analysis of ANGPTL8 (green), MAC-2, α‐SMA, and CD3 (red) and DAPI (blue for nuclei) in AngII-infused AAA in *ApoE^−/−^* mice. *N*=6/group, Data are presented as mean ± SEM, *P<0.05. Scale bars: 50 μm. (**F**) Circulating level of ANGPTL8 in AAA patients. *N*=40/group, Data are presented as mean ± SEM, ***P<0.01. (**G**) Circulating level of ANGPTL8 in AAA mice. *N*=6/group, Data are presented as mean ± SEM, *P<0.05.

To evaluate the involvement of ANGPTL8 in AAA, we examined ANGPTL8 expression in human AAA aortic tissue. Immunohistochemistry results showed that ANGPTL8 protein expression was remarkably higher in human AAA tissues than in normal aortic tissues (*P*<0.05, Figure 1A). We further investigated ANGPTL8 expression in AAA mice model. Immunohistochemistry revealed significantly higher levels of ANGPTL8 in AngII-induced AAA mice compared with controls (*P*<0.01, Figure 1B). Consistent with these results, western blots confirmed that ANGPTL8 expression was significantly higher in AngII-induced AAA mice compared with control mice (*P*<0.05, Figure 1C).

Immunofluorescence staining showed that ANGPTL8 protein colocalized with macrophages and vascular smooth muscle cells (VSMCs) in AAA of humans and mice (Figure 1D,E). In addition, we examined the location of ANGPTL8 in aortic plaques of human and mice. The results of double immunofluorescence staining revealed that ANGPTL8 was highly expressed in macrophages during atherosclerosis (Supplementary Figure S1A,B).

Next, we measured serum ANGPTL8 by ELISA. Circulating ANGPTL8 levels were significantly increased in AAA patients compared with control subjects (402.2 ± 24.51 vs 653.8 ± 20.54 pg/ml; Figure 1F), and the clinical characteristics are shown in Supplementary Table S1. Meanwhile, we measured the levels of serum ANGPTL8 in AAA mice. The results showed serum ANGPTL8 levels were significantly increased in AAA mice (*P*<0.05; Figure 1G) .These results suggest that ANGPTL8 may be involved in the development of AAA.

### ANGPTL8 knockout attenuated AAA and atherosclerosis in ApoE^−/−^ mice

**Figure 2 F2:**
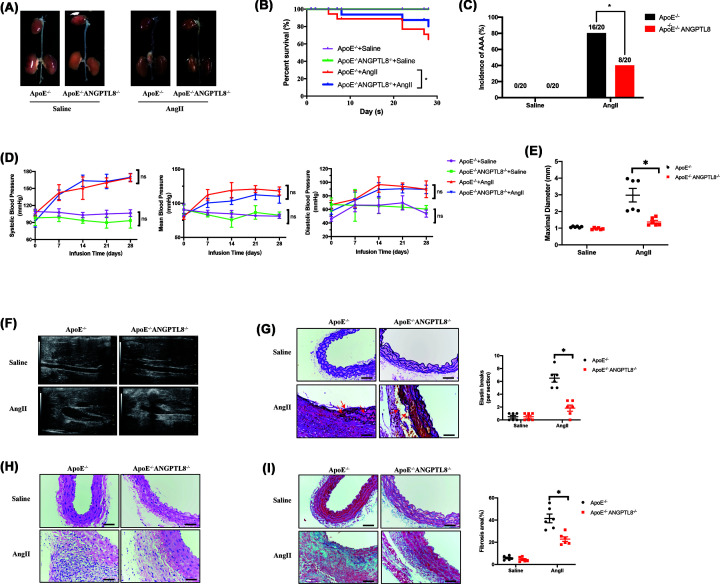
Knockout of ANGPTL8 attenuated AngII-induced AAA in *ApoE^−/−^* mice (**A**) Representative morphology of abdominal aortic specimens in four groups of mice: the ApoE^−/−^ + saline group, the ApoE^−/−^ + AngII group, the ApoE^−/−^ANGPTL8^−/−^ + saline group, and the ApoE^−/−^ANGPTL8^−/−^ + AngII group. (**B**) Survival curve showing the survival of mice at week 4 after saline or AngII administration. (**C**) The incidence of AAA. (**D**) Blood pressure of mice in 4 weeks after saline or AngII administration. (**E**) Quantification of maximal aortic diameter after saline or AngII-infusion for 28 days. Data are presented as means ± SEM. *P<0.05. (**F**) Representative images from ultrasonography of abdominal aortas after saline or AngII-infusion for 28 days. (**G**) Representative images and quantitative analysis of Elastin staining of abdominal aortas in four groups of mice. Scale bars: 50 μm. Data are presented as means ± SEM. *P<0.05. (**H**) Histopathological analysis of representative abdominal aortas by H&E staining and quantification of aortic thickness. Scale bars: 50 μm. Data are presented as means ± SEM. *P<0.05. (**I**) Representative images and quantitative analysis of Masson’s Trichrome staining of abdominal aortas in four groups of mice. Scale bars: 50 μm. Data are presented as means ± SEM. *P<0.05.

To explore the role of ANGPTL8, we bred *ApoE*^−/−^*ANGPTL8*^−/−^ mice to evaluate the effect of ANGPTL8 on AAA and atherosclerosis. There was no observed difference in systolic blood pressure between *ANGPTL8*^−/−^*ApoE*^−/−^ mice and littermate-control *ApoE*^−/−^ mice after AngII infusion (Figure 2D). Serum TG levels were significantly decreased in *ANGPTL8*^−/−^*ApoE*^−/−^ mice with or without AngII infusion (*P*<0.05); however, no differences in serum TC, LDL-C, and GLU levels were observed between *ANGPTL8*^−/−^*ApoE*^−/−^ and *ApoE*^−/−^ mice (*P*>0.05; Supplemental Figure S3A).

Aortic segments from the aortic arch to the bifurcation of the common iliac artery were examined pathologically after AngII infusion (Figure 2A). Kaplan–Meier curves revealed a decrease in survival of *ApoE*^−/−^ mice following AngII infusion, whereas ANGPTL8 knockout increased survival rate (80% vs. 60%, *P*<0.05; Figure 2B). In addition, ANGPTL8 knockout significantly reduced the incidence of AAA (80% vs. 40%, *P*<0.05; Figure 2C) and maximal diameter of the abdominal aorta (1.5 ± 0.2 vs. 3.9 ± 0.1 mm, *P*<0.05) following infusion of Ang II compared with saline (Figure 2E,F). Further pathohistological examination of aortic tissues from mice revealed that elastin breakdown was less severe in AngII-infused *ApoE^−/−^ANGPTL8*^−/−^ mice compared with *ApoE*^−/−^ mice (*P*<0.05; Figure 2G). In addition, AngII-induced increases in wall thickness and fibrosis were significantly ameliorated in *ApoE*^−/−^*ANGPTL8^−/−^* mice (*P*<0.05; Figure 2H,[Fig F2]I).

Furthermore, we used *ApoE*^−/−^*ANGPTL8*^−/−^ mice to evaluate the effect of ANGPTL8 on atherosclerosis. Atherosclerotic lesion formation was assessed after 12 weeks of feeding with a Western diet. Analysis of aortic Oil Red O staining showed significantly reduced lipid accumulation in *ApoE*^−/−^*ANGPTL8*^−/−^ mice compared with *ApoE*^−/−^ mice (*P*<0.05, Supplementary Figure S1C). In addition, Oil Red O and H&E staining of aortic roots showed a significant reduction in plaque areas of *ApoE*^−/−^*ANGPTL8*^−/−^ mice compared with *ApoE*^−/−^ mice (*P*<0.05, Supplementary Figure S1D,E). ANGPTL8 knockout significantly reduced TG levels in atherosclerotic *ApoE*^−/−^ mice, while TC and GLU levels were not significantly different (Supplementary Figure S3B). These results suggest that ANGPTL8 knockout significantly attenuated atherosclerotic lesions in *ApoE*^−/−^ mice.

### ANGPTL8 knockout reduced inflammatory factor expression in AAA and atherosclerosis

**Figure 3:**
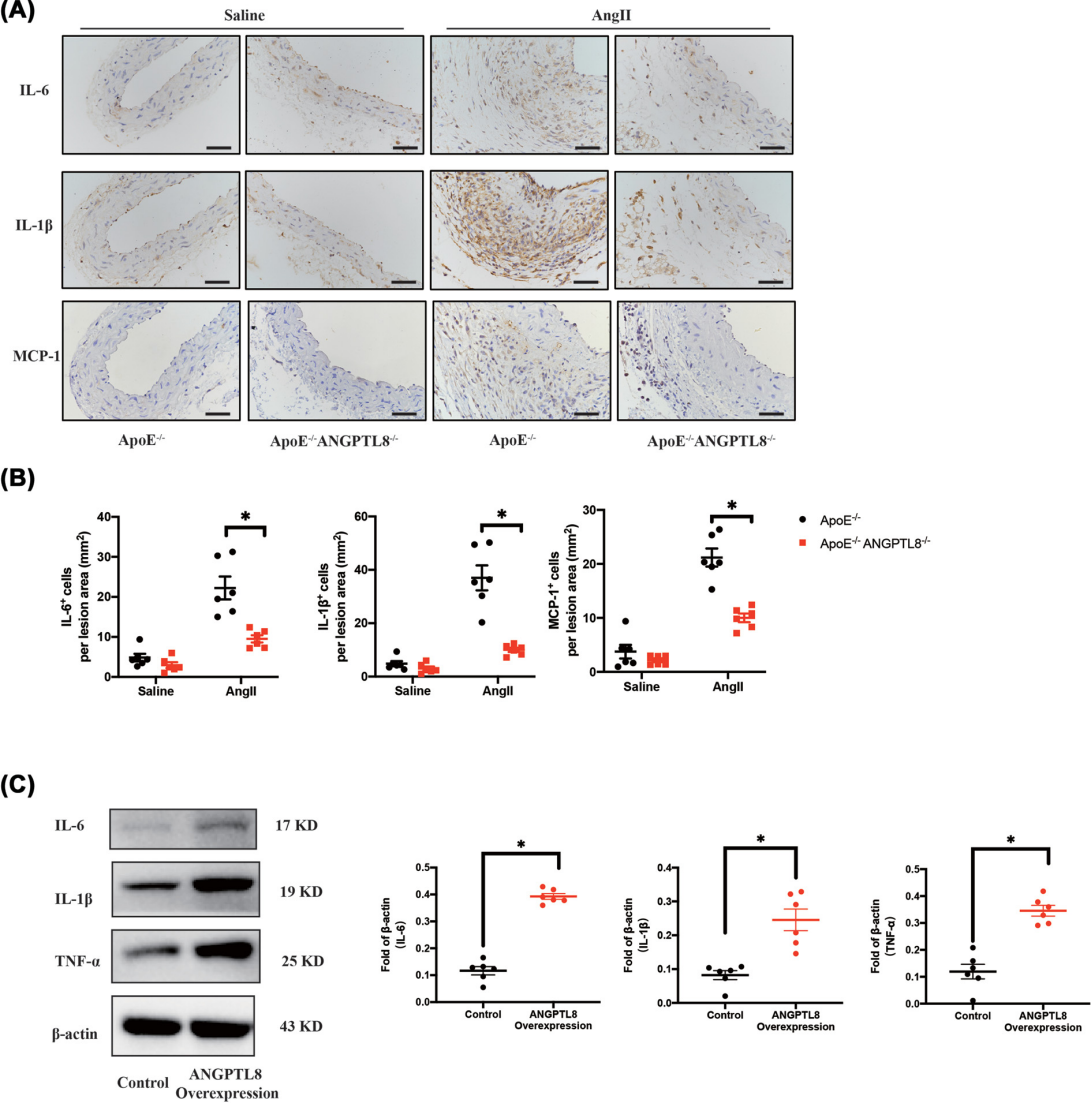
Knockout of ANGPTL8 attenuated inflammation in AngII-induced AAA in *ApoE^−/−^* mice (**A**) Representative images of inflammatory factor IL-6, IL-1β, and MCP-1 staining of abdominal aortas in four groups of mice. Scale bars: 50 μm. (**B**) Quantitative analysis of inflammatory factor IL-6, IL-1β, and MCP-1 staining of abdominal aortas in four groups of mice. *N*=6/group. Data are presented as mean ± SEM. *P<0.05. (**C**) Western blot analysis the expression of IL-6, IL-1β, and TNF-α in RAW264.7. *N*=6/group. Data are presented as mean ± SEM. *P<0.05.

As mentioned above, we found that ANGPTL8 was mainly expressed in macrophages during AAA and atherosclerosis. Therefore, we measured expression of aortic inflammatory cytokines such as interleukin 6 (IL-6), IL-1β, and monocyte chemotactic protein 1 (MCP-1). In mice, immunohistochemical analysis showed that AngII increased expression of IL-6, IL-1β, and MCP-1 in *ApoE*^−/−^ compared with saline, and ANGPTL8 deletion reduced AngII-induced up-regulation of IL-6, IL-1β, and MCP-1 (*P*<0.05; Figure 3A,B). Similarly, we observed expression of IL-6 and MCP-1 in aortic plaques of *ApoE*^−/−^*ANGPTL8*^−/−^ mice (*P*<0.05, Supplementary Figure S2A). Collectively, these results revealed that expression of inflammatory factors was significantly decreased in ANGPTL8-knockout mice. Next, we used RAW264.7 cells for our *in-vitro* experiments. Western blot showed that ANGPTL8 overexpression increased expression of IL-6, IL-1β, and TNF-α (*P*<0.05, Figure 3C). Collectively, these results support ANGPTL8-overexpression exacerbated secretion of proinflammatory factors of macrophages.

### ANGPTL8 knockout reduced expression of MMP-2 and MMP-9 in AAA and atherosclerosis

**Figure 4:**
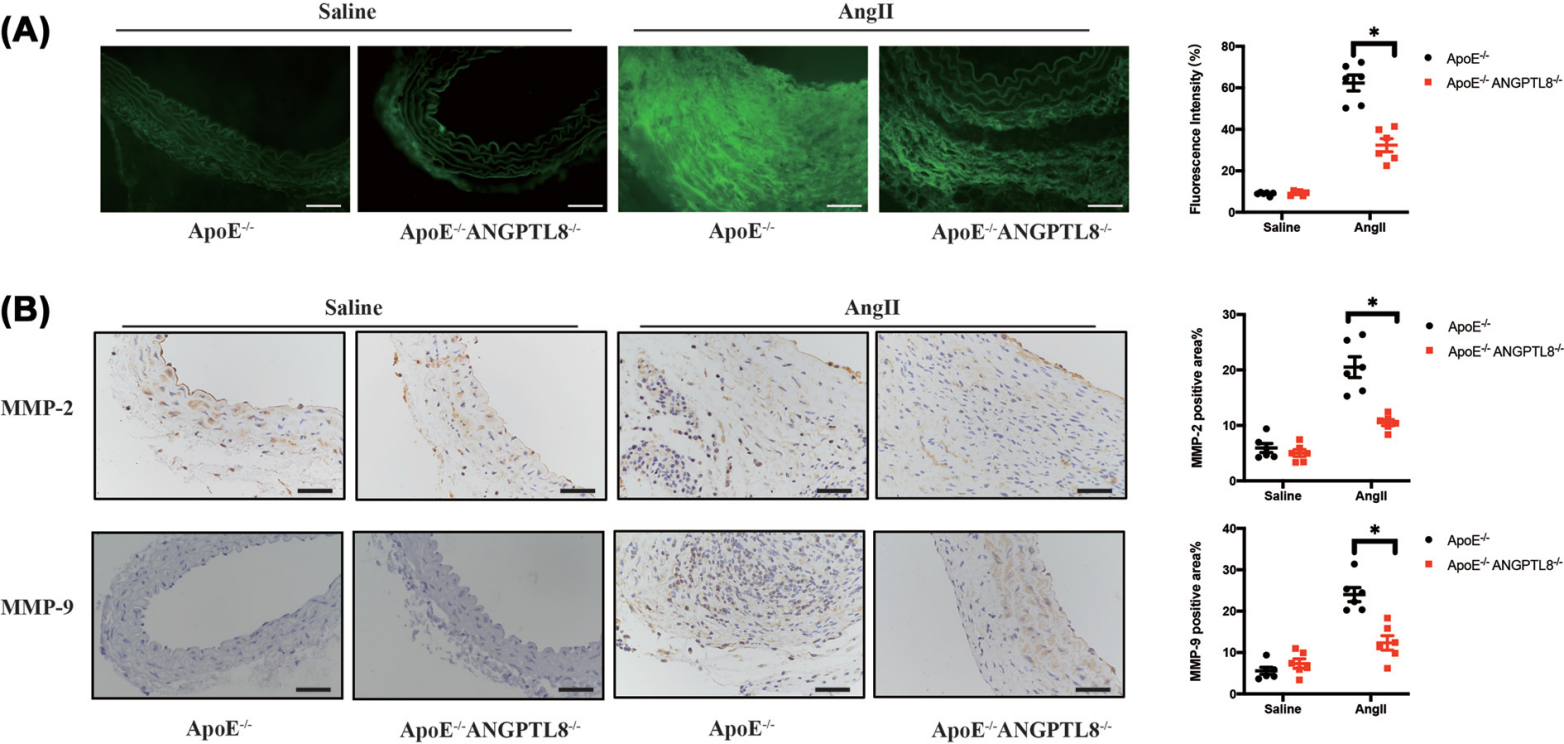
Knockout of ANGPTL8 attenuated MMPs in AngII-induced AAA in *ApoE^−/−^* mice (**A**) Representative photomicrographs and quantitative analysis of *in-situ* zymography of abdominal aortas in four groups of mice. Scale bars: 50 μm. (**B**) Representative images and quantitative analysis of MMP-2 and MMP-9 staining of abdominal aortas in four groups of mice. *N*=6/group. Data are presented as mean ± SEM. *P<0.05. Scale bars: 50 μm.

MMP-2 and MMP-9 contribute to the pathogenesis of aortic aneurysm by cleaving the extracellular matrix of blood vessels. *In-situ* zymography showed that AngII infusion increased the activity of MMPs, whereas ANGPTL8 deletion could reduce it (*P*<0.05; Figure 4A). We evaluated expression of MMP-2/9 in the mouse aorta using immunohistochemistry. We found that ANGPTL8 knockout decreased expression of MMP-2 and MMP-9 (*P*<0.05; Figure 4B). Extracellular matrix remodeling is also an important pathological change associated with atherosclerosis. Observation of MMP expression in aortic plaques of mice revealed that expression of MMP-2 and MMP-9 was significantly decreased in ANGPTL8-knockout mice (*P*<0.05, Supplementary Figure S2B).

### ANGPTL8 knockout reduced VSMC apoptosis in AAA

**Figure 5:**
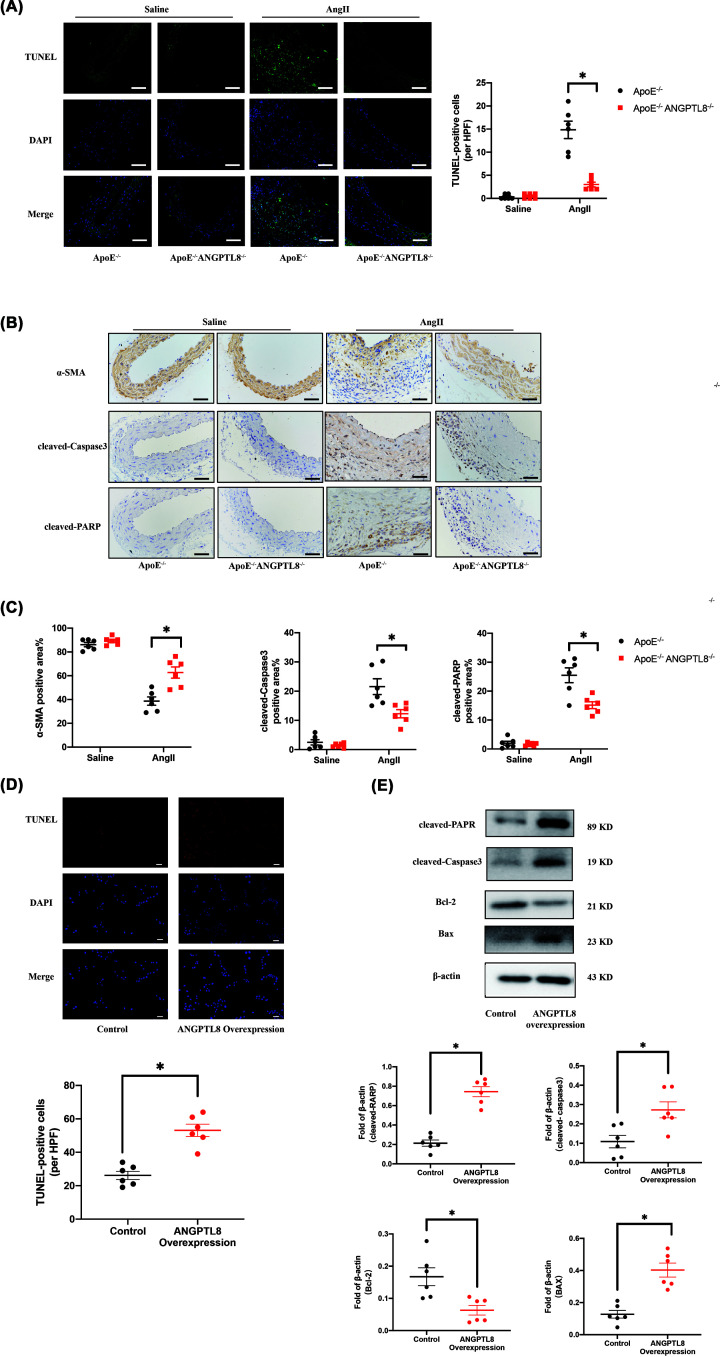
Knockout of ANGPTL8 attenuated apoptosis in AngII-induced AAA in *ApoE^−/−^* mice (**A**) TUNEL staining of the abdominal aortas in four groups of mice. Scale bars: 50 μm. Data are presented as means ± SEM. *P<0.05. (**B**) Representative images of α-SMA, cleaved-Caspase 3, and cleaved-PARP staining of abdominal aortas in four groups of mice. Scale bars: 50 μm. (**C**) Quantitative analysis of α-SMA, cleaved-Caspase 3, and cleaved-PARP staining of abdominal aortas in four groups of mice. *N*=6/group. Data are presented as mean ± SEM. *P<0.05. (**D**) TUNEL staining of HASMCs. Scale bars: 50 μm. N = 6/group. Data are presented as means ± SEM. *P<0.05. (**E**) Western blot analysis the expression of cleaved-PARP, cleaved-caspase 3, Bcl-2, and Bax in HASMCs. *N*=6/group. Data are presented as mean ± SEM. *P<0.05.

Apoptosis of VSMCs is one of the main pathogenic features of aortic aneurysm [18]. We found that ANGPTL8 knockout reduced VSMC apoptosis assessed by TUNEL staining in the aortic wall of mice (*P*<0.05; Figure 5A). Consistently, immunohistochemistry results showed that AngII infusion reduced expression of α-SMA in the abdominal aortic wall, while ANGPTL8 knockout significantly decreased the loss of VSMC (*P*<0.05; Figure 5B). Immunohistochemical analysis revealed that ANGPTL8 knockout reduced the elevation of cleaved-PARP and cleaved-caspase 3 levels induced by AngII in *ApoE*^−/−^ mice (*P*<0.05; Figure 5C). These results suggest that ANGPTL8 deletion could protect against AngII-induced AAA by reducing VSMCs apoptosis. We used HASMCs to observe the effect of ANGPTL8 overexpression on apoptosis. Western blot showed that ANGPTL8 overexpression increased expression of cleaved-PARP, cleaved-Caspase3, and BAX; while decreased expression of Bcl-2 (*P*<0.05, Figure 5E). Collectively, these results support ANGPTL8-overexpression exacerbated apoptosis of VSMCs.

### Inhibition of ANGPTL8 significantly reduced AAA in *ApoE^−/−^* mice

**Figure 6:**
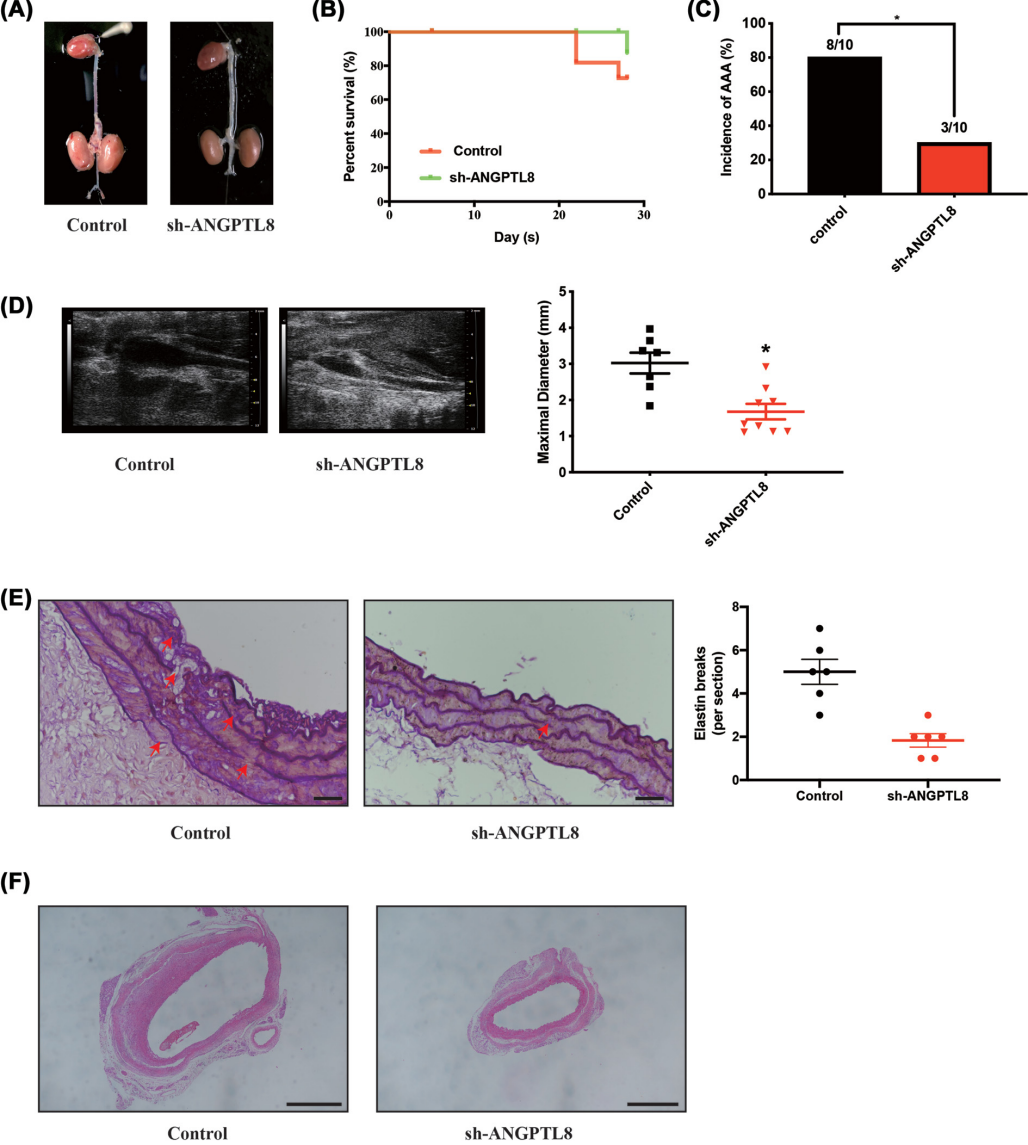
Inhibition of ANGPTL8 attenuated AngII-induced AAA in *ApoE^−/−^* mice (**A**) Representative morphology of abdominal aortic specimens in Control and sh-ANGPTL8 groups of mice. (**B**) Survival curve of Control and sh-ANGPTL8 groups of mice. (**C**) The incidence of AAA. (**D**) Representative images from ultrasonography and quantification of maximal aortic diameter after AngII-infusion for 28 days. Data are presented as means ± SEM. *P<0.05. (**E**) Representative images and quantitative analysis of H&E staining of abdominal aortas in Control and sh-ANGPTL8 groups of mice. Scale bars: 50 μm. Data are presented as means ± SEM. *P<0.05. (**F**) Representative images and quantitative analysis of Elastin staining of abdominal aortas in Control and sh-ANGPTL8 groups of mice. Scale bars: 50 μm. Data are presented as means ± SEM. *P<0.05.

To further confirm the protective effect elicited by ANGPTL8 inhibition of AAA, we inhibited ANGPTL8 expression using sh-RNA intervention in *ApoE*^−/−^ mice. ANGPTL8-knockdown efficiency were confirmed by western blot analysis of liver tissues (Supplementary Figure S4). Aortic segments from the aortic arch to the bifurcation of the common iliac artery were examined pathologically after AngII infusion (Figure 6A). Kaplan–Meier curves showed decreased survival of *ApoE*^−/−^ mice following AngII infusion, whereas ANGPTL8 sh-RNA increased survival (90% vs. 70%, *P*<0.05; Figure 6B). Following Ang II infusion, ANGPTL8 sh-RNA significantly reduced the incidence of AAA (30% vs. 80%, *P*<0.05) and maximal diameter of the abdominal aorta (*P*<0.05) compared with *ApoE*^−/−^ controls (Figure 6C,D). Further pathohistological examination of aortic tissues from mice revealed that the elastin breakdown was less severe in sh-ANGPTL8 mice compared with *ApoE^−/−^* mice after AngII infusion (*P*<0.05; Figure 6E). In addition, knockdown of ANGPTL8 mice significantly attenuated AAA progression in AngII-induced AAA (Figure 6F).

## Discussion

The present study is the first to demonstrate a role for ANGPTL8 in AngII-induced AAA formation and verify a direct role of ANGPTL8 in atherosclerotic plaque progression of *ApoE*^−/−^*ANGPTL8*^−/−^ mice. Specifically, we found that ANGPTL8 knockout attenuated the occurrence and development of AngII-induced AAA and attenuated atherosclerotic plaque progression in *ApoE*^−/−^ mice.

ANGPTL8 is an atypical member of the ANGPTLs and studies showed that ANGPTLs was associated with AAA. Molecular fingerprint for AAA showed ANGPTL4 is positively associated with progression of larger AAA, and with rupture [19]. Previous studies have demonstrated that ANGPTL2 expression is increased in macrophages in tissues of patients with AAAs and that macrophage-derived ANGPTL2 is involved in the development of AAAs via triggering inflammatory responses in the vessel wall and extracellular matrix degradation [20]. But ANGPTL8 role in the AAA has barely been explored.

In recent years, ANGPTL8 has attracted attention for its involvement in the development of CVD [13,21]. Studies have shown that serum ANGPTL8 levels are positively correlated with the severity of coronary artery disease (CAD) and peripheral arterial disease [22]. Our previous study demonstrated that plasma ANGPTL8 levels were significantly higher in patients with CAD compared with controls in Chinese nondiabetic individuals, and levels of ANGPTL8 were positively associated with CAD severity [23]. Recently, we published a study showing an association between ANGPTL8 and the occurrence and development of thoracic aortic dissection (TAD). Serum ANGPTL8 levels were significantly elevated in patients with TAD, and positively correlated with TAD diameter [8]. ANGPTL8 knockout can prevent chronic intermittent hypoxia-induced aortic vascular remodeling [11].

Atherosclerosis represents an important independent risk factor for AAA, as people with AAA often have atherosclerosis [24]. AAA shares many of pathological processes with atherosclerosis [25]. We found previously that treatment with adeno-associated virus vectors containing small hairpin (shRNA) against ANGPTL8 attenuated atherosclerosis formation in *ApoE^−/−^* mice [15]. But the direct role of ANGPTL8 in the atherosclerosis has not been demonstrated. In the present study, our data suggest that ANGPTL8 knockout attenuated atherosclerosis, we further verified the direct role of ANGPTL8 in atherosclerotic plaque progression of *ApoE^−/−^ANGPTL8^−/−^* mice.

Inflammation plays an important role in the formation of atherosclerotic plaques [26]. Indeed, inflammation of the vessel wall is present throughout the pathogenesis of AAA [27]. Inflammatory cells produce abundant MMPs and reactive oxygen species that degrade extracellular matrix proteins of the vessel wall and promote apoptosis of vascular smooth muscle, causing thinning of the mesothelium and dilatation of the vessel wall into aneurysm when it cannot tolerate the pressure of blood flow [4]. The relationship between ANGPTL8 and inflammation has been confirmed by many studies. In patients with type 2 diabetes mellitus, serum ANGPTL8 levels were positively correlated with the degree of inflammation and oxidative stress [10]. ANGPTL8 plays a role in tumor necrosis factor‐α-induced inflammation and extracellular matrix degradation in nucleus pulposus cells [28]. Moreover, plasma ANGPTL8 levels were significantly elevated in patients diagnosed with severe infections [9]. In mice, there is a significant correlation between circulating ANGPTL8 levels and the acute inflammatory response induced by lipopolysaccharide [9]. Our previous study found that ANGPTL8 knockdown ameliorated atherosclerosis in *ApoE*^−/−^ mice, whereas ANGPTL8 overexpression promoted plaque formation. Notably, ANGPTL8 contributes to macrophage foam cell formation [15]. The results of the present study show that ANGPTL8 knockout inhibited expression of inflammatory factors in atherosclerotic plaque tissue in high-fat chow-fed *ApoE*^−/−^ mice and suppressed the atherosclerotic plaque process. Notably, ANGPTL8 was significantly up-regulated in human and mouse AAA tissues. Thus, we next investigated the role of ANGPTL8 in AAA formation for the first time using *ANGPTL8*^−/−^*ApoE*^−/−^ mice. ANGPTL8 knockout elicited a protective effect, including attenuated expression of inflammatory factors in AAA aortic tissues, significantly reduced incidence of AAA, and significantly reduced aortic diameters in AngII-infused *ApoE*^−/−^ mice.

ANGPTL8 regulates various cell processes, including proliferation and apoptosis. ANGPTL8 attenuated the apoptosis of pancreatic cancer cell by down-regulating Bcl-2 [29]. ANGPTL8 may act as a moderate suppressor of hepatocellular carcinoma cell proliferation by affecting Wnt signaling modulators [12]. Our immunohistochemistry results revealed that AngII reduced VSMCs in AAA, whereas ANGPTL8 deletion increased VSMCs in mice. Apoptosis of VSMCs was shown to be markedly increased in aortic aneurysmal tissues; however, ANGPTL8 deletion decreased apoptosis in mice. A previous study from our laboratory suggested that ANGPTL8 siRNA could attenuate VSMC apoptosis [8]. The effect of ANGPTL8 on VSMC apoptosis may play a role in the development of AAA.

ANGPTL8 plays an important role in lipid metabolism. Although many clinical studies have shown that ANGPTL8 is associated with the levels of circulating TG, TC, LDL-C and HDL-C, in ANGPTL8-knockout mice, only plasma TG levels are significantly reduced, while cholesterol levels are unaltered [30]. Previous studies have shown that serum TG levels in ANGPTL8-knockout mice were one-third of those in wide-type mice, and ANGPTL8 overexpression in mice increased serum TG levels [31]. Gusarova et al. [32] have reported REGN3776 is a humanized monoclonal antibody that binds monkey and human ANGPTL8 with high affinity. REGN3776 decreased mice plasma TGs and increased lipoprotein lipase activity. Single administration of REGN3776 normalized plasma TGs in dyslipidemic cynomolgus monkeys. ANGPTL8 monoclonal antibodies have great potential for clinical use in lipid metabolism diseases. In the present study, we found that the levels of circulating TG were lower in *ANGPTL8^−/−^ApoE^−/−^* mice compared with the *ApoE^−/−^* mice. This suggests that ANGPTL8 promotes atherosclerotic CVD partly by increasing plasma TG.

Despite our novel and significant findings, there are several limitations of the present study. First, we observed the expression of ANGPTL8 in AAA patients; we used aortas obtained from healthy transplant donors as Control aortic specimens, this represents a different part of the aorta (aortic root vs .abdominal aorta); Second, we only used male mice because of the high success rate and good reproducibility in this model. However, certain cardiovascular characteristics, such as blood pressure, serum lipid profile, endothelial function, and abundance of thrombotic plaques differ between females and males. Third, the mechanisms underlying how ANGPTL8 affects atherosclerosis and AAA need further investigation.

## Conclusion

In conclusion, we found that ANGPTL8 knockout significantly inhibited the progression of AAA and atherosclerosis in *ApoE*^−/−^ mice. Thus, inhibition of ANGPTL8 may provide a new target for the treatment of atherosclerotic CVD.

## Clinical perspectives

- AAA, as a life‐threatening disease, can be attributed to atherosclerosis. Effective pharmacological therapies to inhibit AAA expansion or prevent aneurysm rupture have not been established clinically, and the molecular pathogenesis of AAA remains unclear. There is an urgent clinical need to find targets to inhibit the development of AAA.
- Our studies found that ANGPTL8 was significantly up-regulated in AAA tissues of human and experimental mice. Knockout of ANGPTL8 significantly reduced AngII-induced AAA formation, vascular elastin dissection, aortic inflammatory cytokines, matrix metalloproteinase expression, and smooth muscle cell apoptosis in *ApoE^−^/^−^* mice. Moreover, atherosclerotic plaque lesions were significantly inhibited in *ApoE^−^/^−^*ANGPTL8^−^/^−^ mice and expression of aortic inflammatory factors and matrix metalloproteinases was significantly decreased. Similarly, ANGPTL8 shRNA also significantly reduced AngII-induced AAA formation in *ApoE^−^/^−^* mice.
- ANGPTL8 knockout significantly inhibited the progression of AAA and atherosclerosis in *ApoE^−^/^−^* mice. Thus, inhibition of ANGPTL8 may provide a new target for the treatment of AAA.

## Supplementary Material

Supplementary Figures S1-S4 and Table S1

## Data Availability

All supporting data for the present manuscript are included in the figures and the accompanying Supplementary Files.

## Abbreviations

AAA: abdominal aortic aneurysm
AngII: angiotensin II
ANGPTL8: angiopoietin-like protein 8
CAD: coronary artery disease
CVD: cardiovascular disease
GLU: glucose
HASMC: human aortic smooth muscle cell
IL-1β: interleukin 1β
IL-6: interleukin 6
LDL-C: low-density lipoprotein cholesterol
MCP-1: monocyte chemotactic protein 1
MMP: matrix metallopeptidase
PARP: poly-ADP-ribose polymerase
SEM: standard error of the mean
shRNA: short hairpin RNA
TAD: thoracic aortic dissection
TC: total cholesterol
TG: triglycerides
VSMC: vascular smooth muscle cell
HE: hematoxylin-eosin staining
PCR: polymerase chain reaction
