# Supplementary material for: ANGPTL8 deletion attenuates abdominal aortic aneurysm formation in ApoE−/− mice
Source: Clin Sci (Lond). 2023 Jun 28;137(12):979–93. doi: 10.1042/CS20230031 (PMC10311111; doi:10.1042/CS20230031)
Supplement: Supplementary Figures S1-S4 and Table S1 [file CS-2023-0031_supp.pdf]

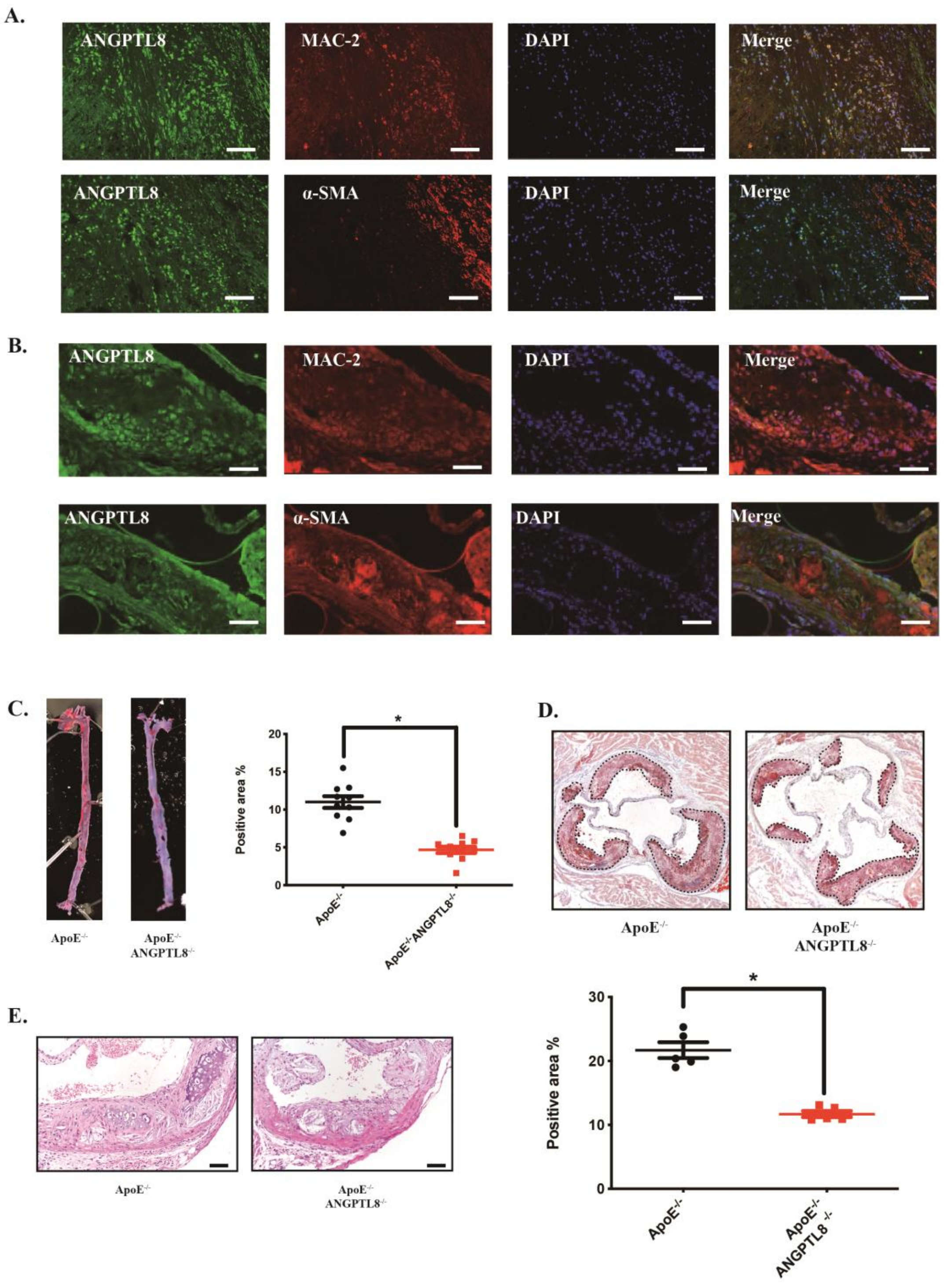

Supplementary figure 1. Knockout of ANGPTL8 attenuated atherosclerosis in *ApoE$^{-/-}$* mice. (A) Immunofluorescence analysis of ANGPTL8(green), MAC-2 or α-SMA(red) and DAPI (blue for nuclei) in human atherosclerotic lesions. **(B)** Immunofluorescence analysis of ANGPTL8(green), MAC-2 or α-SMA(red) and DAPI (blue for nuclei) in mice atherosclerotic lesions. N = 6/group, Data are presented as

mean ± SEM, *P < 0.05. Scale bars: 50 μm. **(C)** En face lesion area representative images and analysis of aortae from *ApoE*$^{-/-}$ mice after high-fat diet. **(D)** Representative images and analysis for Oil red O staining in the aortic roots of *ApoE*$^{-/-}$ and *ApoE*$^{-/-}$*ANGPTL8*$^{-/-}$ mice after high-fat diet. **(E)** HE staining of aortic roots of *ApoE*$^{-/-}$ and *ApoE*$^{-/-}$*ANGPTL8*$^{-/-}$ mice after high-fat diet. Scale bars: 100 μm.

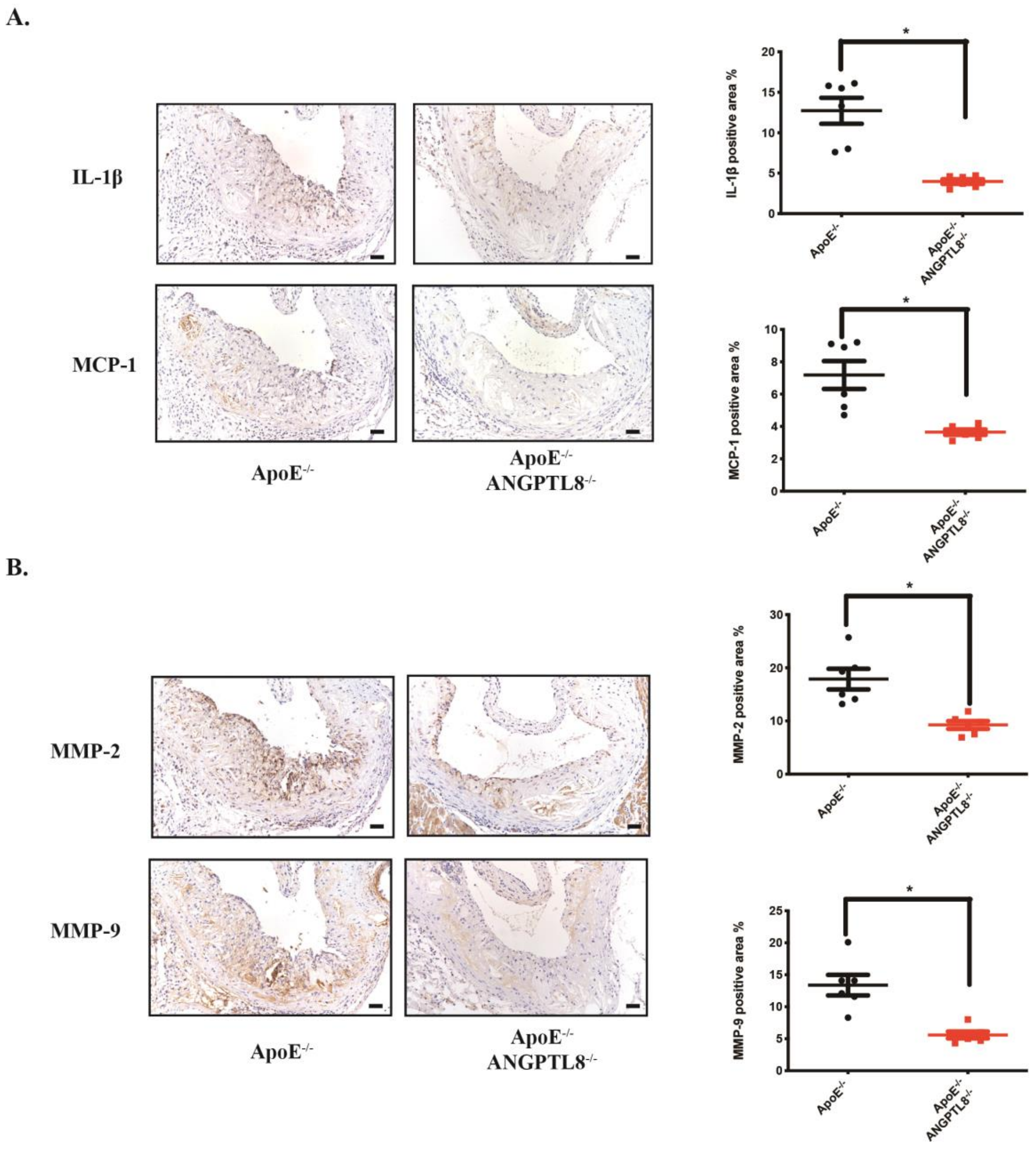

Supplementary figure 2. Knockout of ANGPTL8 attenuated inflammation and MMPs in atherosclerosis in *ApoE*$^{-/-}$ mice. (A) Representative images and quantitative analysis of IL-1β and MCP-1 staining of aortic roots of *ApoE*$^{-/-}$ and *ApoE*$^{-/-}$*ANGPTL8*$^{-/-}$ mice after high-fat diet. N = 6/group, Data are presented as mean ± SEM. *P < 0.05. Scale bars: 50 μm. **(B)** Representative images and quantitative analysis of MMP-2 and MMP-9 staining of aortic roots of *ApoE*$^{-/-}$ and *ApoE*$^{-/-}$*ANGPTL8*$^{-/-}$ mice after high-fat diet. Scale bars: 50 μm. N = 6/group, Data are presented as mean ± SEM. *P < 0.05.

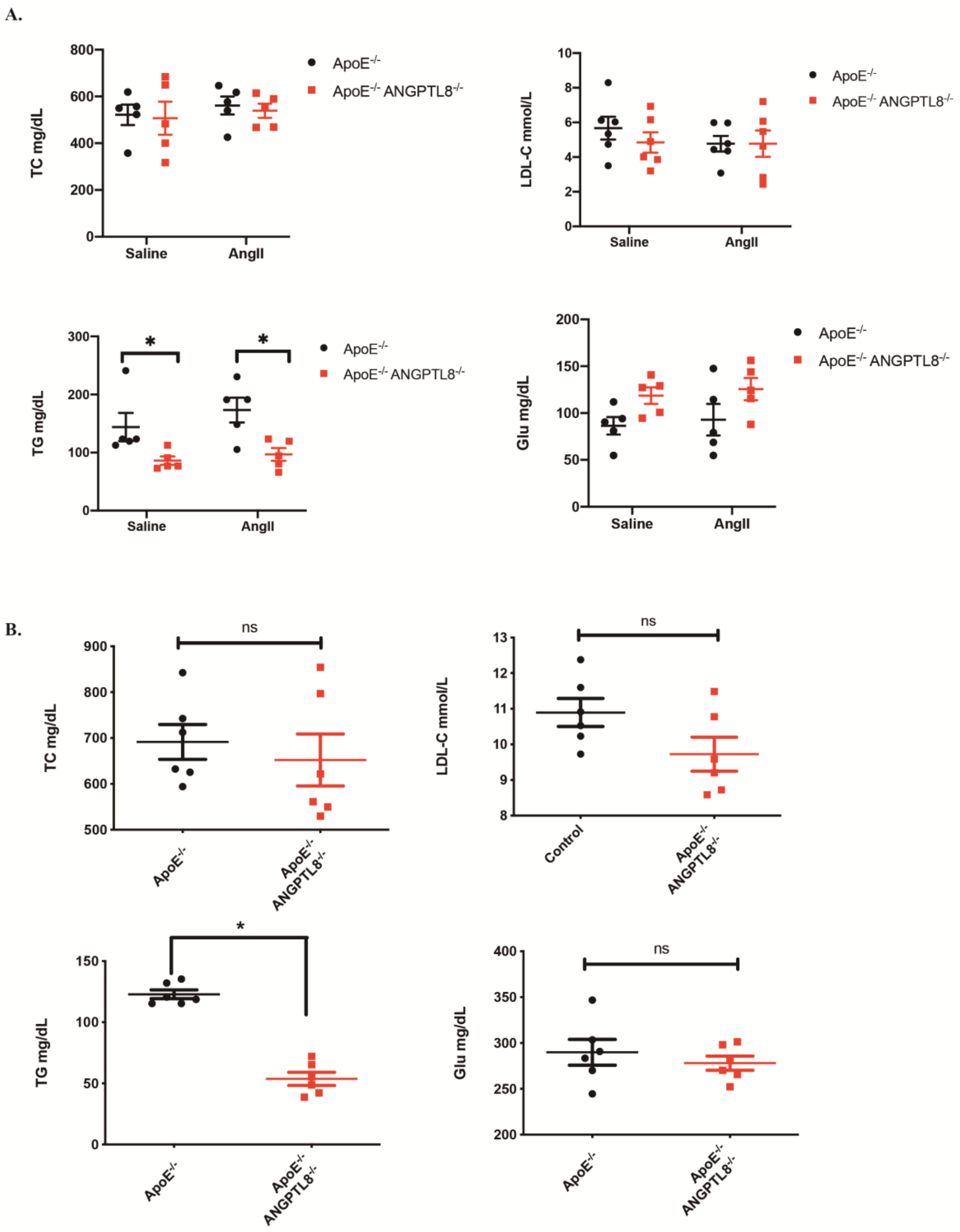

Supplementary figure 3. Knockout of ANGPTL8 decreases the plasma levels of triglycerides (TG) instead of low-density lipoprotein cholesterol (LDL-C), total cholesterol (TC) and glucose (GLU). (A) Measurement of TC, LDL-C, TG and GLU in 4 groups of mice: the *ApoE*$^{-/-}$ +saline group, the *ApoE*$^{-/-}$+Ang II group, the

ApoE$^{-/-}$*ANGPTL8*$^{-/-}$+saline group, and the *ApoE*$^{-/-}$*ANGPTL8*$^{-/-}$+Ang II group. **(B)** Measurement of TC, LDL-C, TG and GLU in *ApoE*$^{-/-}$ and *ApoE*$^{-/-}$*ANGPTL8*$^{-/-}$ mice after high-fat diet. N = 6/group, Data are presented as means ± SEM. *$p < 0.05$.

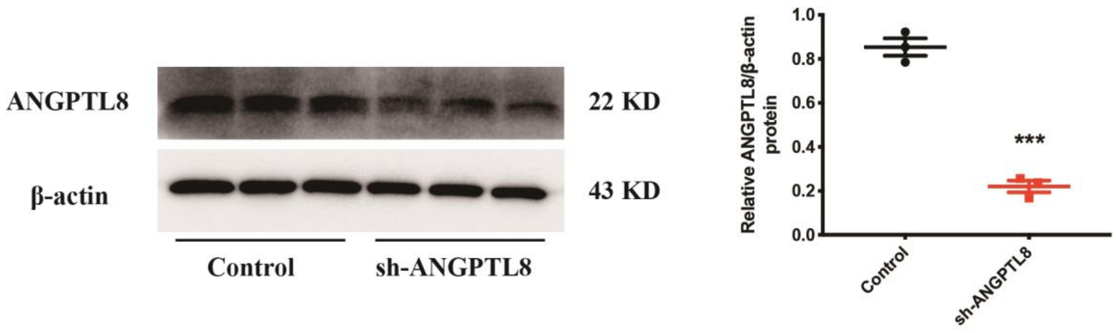

Supplementary figure 4. Western blotting of liver tissues in sh-ANGPTL8 mice.

Supplemental Table 1. Anthropometric and biochemical characteristics of the subjects included in the study.

	Control N=40	AAA N=40	P
Age (years)	54.1 ± 11.48	52.65 ± 15.73	0.639
Male (n,%)	34 (85%)	32 (80%)	0.352
BMI (kg/m^2)	26.86 ± 2.48	25.40 ± 2.47	0.029*
Smoker (n,%)	6 (15%)	14 (35%)	0.001*
Drinker (n,%)	6 (15%)	4 (10%)	0.285
Hypertension (n,%)	4 (10%)	26 (65%)	<0.001**
Diabetes(n,%)	4 (10%)	2 (5%)	0.180
SBP (mmHg)	132.20 ± 21.70	123.50 ± 14.94	0.050
DBP (mmHg)	79.36 ± 14.43	73.93 ± 10.71	0.076
TG (mmol/L)	1.77 (1.24 – 1.91)	1.65 (0.99 – 1.91)	0.623
TC (mmol/L)	4.07 ± 0.79	3.62 ± 1.13	0.057
Hs-CRP (mmol/L)	4.91 ± 7.55	12.64 ± 8.71	<0.001**
D-Dimer (ug/mL)	95.05 (38.5 – 88.5)	1849.00 (597.50 – 2915.00)	<0.001**
ANGPTL8 (pg/mL)	402.20 ± 155.0	653.80 ± 129.9	<0.001**
Aortic diameter (mm)	19.72 ± 2.19	52.56 ± 16.94	<0.001**
Use of ace-inhibitor	4 (10%)	12 (30%)	<0.001**
Use of statins	14 (35%)	12 (30%)	0.450
Use of b-blocker	6 (15%)	28 (70%)	<0.001**
Use of antiplatelets	18 (45%)	24 (60%)	0.034*

Results are expressed as mean±standard deviation, median (interquartile range) or n (%). Differences between groups were analyzed by the independent Student t-test, χ^2 text, or Wilcoxon test.

Abbreviations: BMI, body mass index; SBP, systolic blood pressure; DBP, diastolic blood pressure; TG, triglycerides; TC, total cholesterol; Hs-CRP, high-sensitivity C-reactive protein; .

*P<0.05, **P<0.001.
